# Cobalt Complexes as Antiviral and Antibacterial Agents

**DOI:** 10.3390/ph3061711

**Published:** 2010-05-26

**Authors:** Eddie L. Chang, Christa Simmers, D. Andrew Knight

**Affiliations:** 1 Center for Bio/Molecular Science and Engineering, Naval Research Laboratory, 4555 Overlook Avenue S.W., Washington, DC 20375, USA; 2 Chemistry Department, Florida Institute of Technology, 150 West University Boulevard, Melbourne, FL 32901, USA; E-Mail: simmersc2008@my.fit.edu (C.S.)

**Keywords:** antiviral, antibacterial, cobalt

## Abstract

Metal ion complexes are playing an increasing role in the development of antimicrobials. We review here the antimicrobial properties of cobalt coordination complexes in oxidation state 3+. In addition to reviewing the cobalt complexes containing polydentate donor ligands, we also focus on the antimicrobial activity of the homoleptic [Co(NH_3_)_6_]^3+^ ion.

## 1. Introduction

Metals have been used in the treatment of diseases of humans since ancient times. The Chinese were using elemental gold for the treatment of diseases, a practice known as chrysotherapy, as far back as 2500 BC [1]. In more recent times, a stable metal coordination complex based on the element platinum, *cis*-[PtCl_2_(NH_3_)_2_] (cisplatin), has become the most well known of all metal based drugs and hundreds of articles have been published on the synthesis and activity of complexes derived from the parent cisplatin molecule. The mechanism of action of cisplatin at the molecular level, involving interaction of the labile Pt(II) ion with DNA, is included in undergraduate inorganic chemistry curricula in the United States, and introductory college level inorganic textbooks describe the coordination chemistry of Pt(II) with nitrogen containing bases of nucleic acids, and use cisplatin to illustrate the hard-soft relationship between metal ions and ligand donor atoms. Since Rosenberg's initial discovery of cisplatin in 1969 [2], many more examples of metal-containing drugs have been reported in the literature. Gold containing complexes such as auranofin are commonly used to treat rheumatoid arthritis [3], radiopharmaceuticals based on metals such as technetium and rhenium are used in imaging and radiotherapy [4], and ruthenium complexes have had some success as anticancer drugs [5]. Complexes containing gadolinium, cobalt, lithium, bismuth, iron, calcium, lanthanum, gallium, tin, arsenic, rhodium, copper, zinc, aluminum and lutetium have all been used in medicine [6]. More recently, cobalt(III) based ligand complexes have been found to possess both antiviral and antibacterial activities. We review here the current status of the biological activities of Co(III) complexes formed with mono and polydentate ligands.

## 2. Cobalt(III) Ions in Biological Systems

Only a small number of cobalt(III) complexes are known to have biochemical roles. Vitamin B_12_ is a cobaloxime, a cobalt complex containing a glyoxime ligand, and is one of the rare examples of a naturally occurring organometallic complex *i.e.* possessing a metal carbon bond. The structure has been solved by X-ray crystallography. The Co^3+^ ion in vitamin B_12_ is stabilized by a chelating tetradentate macrocycle known as a corrin in which the four nitrogen atoms are located in equatorial positions in the octahedral geometry. The remaining ligands in the axial positions are a labile adenosyl residue in which the 5' carbon is directly bonded to cobalt (being labile, the ligand coordinated in this position is variable), and an *N*-bonded dimethylbenzimidazole ligand. Vitamin B_12_ is a cofactor for a number of enzymes, virtually all of which are isomerases, methyl transferases or dehalogenases. Other examples of cobalt containing enzymes in biology include nitrile hydratase, prolidase, glucose isomerase, methylmalonyl-CoA carboxytransferase, aldehyde decarbonylase, lysine-2,3-aminomutase, bromoperoxidase and methionine aminopeptidase but only nitrile hydratase possesses cobalt in oxidation state 3+ [7]. Co(III) is also found in certain cobalt-porphyrin containing proteins [8].

## 3. Antiviral Activity of Cobalt(III) Complexes

**Figure 1:**
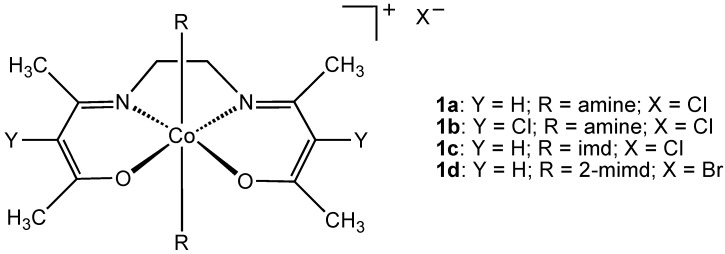
Structure of CTC-type cobalt(III) complexes (imd = imidazole, 2-mimd = 2-methylimidazole).

The simple Co^3+^ ion is unstable in water, but can be stabilized against reduction to Co^2+^ by coordination to ligands or chelators. By far the most common ligand type used to stabilize the cobalt(III) ion in aqueous solution is the chelating N,O donor ligand. Surprisingly, cobalt(III) complexes derived from this ligand donor set have found application as antibacterial or antiviral agents. One of the most promising classes of Co(III) complexes containing N, O donor ligands is the CTC series of complexes **1** based on a chelating Schiff base (imd = imidazole; 2-mimd = 2-methylimidazole) (Figure 1). In 1998, Epstein and coworkers reported that the cobalt complex CTC-96 (**1d**) was effective in the treatment of epithelial herpetic keratitis, one of the major causes of blindness in industrial nations [9]. Studies using the CTC class of drugs were performed using a rabbit eye model infected with Herpes Simplex Virus Type 1 (HSV-1) and all complexes inhibited HSV-1 replication in vitro with as little as 5 μg/mL required for strong antiviral activity.

**Figure 2:**
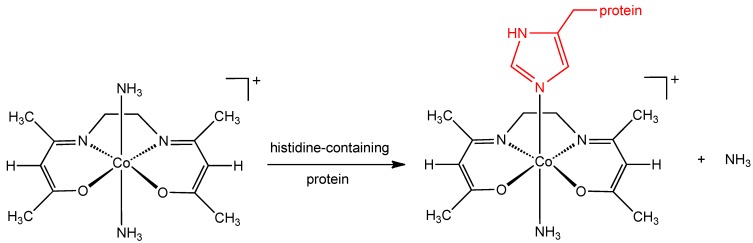
Proposed interaction of histidine containing protein with CTC-complexes.

Although the mechanism of action of the CTC class of complexes has not been completely elucidated, it has been suggested that the molecular target is the herpes virus maturational protease, a serine protease containing large amounts of the amino acid, histidine. CTC complexes are known to bind strongly to the histidine molecule and the stability of Co(III) complexes with histidine in the axial position is particularly high (Figure 2) [10]. Supporting evidence for the involvement of this axial histidine interaction comes from the observation that a cobalt(III) chelate complex with an imidazole ligand already in the axial position (**1c**, CTC-82), is inactive against HSV-1. There is evidence that CTC-96 inhibits membrane fusion events preventing virus entry, CTC-96 inhibited plaque formation by VSV (vesicular stomatitis virus) and VZV (varicella-zoster virus) [11]. In 2006 Epstein reported the activity of CTC-96 (**1d**) against adenovirus in a cell culture model and also against adenovirus keratoconjunctivitis in a rabbit model [12]. The synthesis of CTC-96 was described by Böttcher *et al.* [13] and the drug formulation was developed and sold as Doxovir^TM^; by the Redox Pharmaceutical Corporation [14]. CTC compounds were also shown to inhibit Sp1, a DNA binding zinc finger protein and this has potential implications for the use of CTC compounds in the treatment of human immunodeficiency virus type 1 (HIV-1) [15]. The CTC-23 complex (**1a**) has superoxide scavenging properties [16].

Hexamminecobalt(III) chloride, [Co(NH_3_)_6_]Cl_3_ (**2**, "Cohex") is an example of a classical Werner complex. Known for over a century, it is thermally and kinetically stable in aqueous solution and is easily synthesized. Cohex is a commercially available compound in which six ammonia ligands are arranged in an octahedral geometry about the Co(III) ion. In contrast to the CTC series of complexes, in which the axial ligands are readily exchanged for histidine ligand, the ammonia ligands on Cohex are inert towards ligand exchange [17]. Despite the fact that Cohex lacks the ability to coordinate to, and hydrolyze phosphodiester bonds, it does possess the ability to hydrogen bond with the nitrogenous bases of nucleotides and the phosphate backbone of DNA [17].

**Figure 3:**
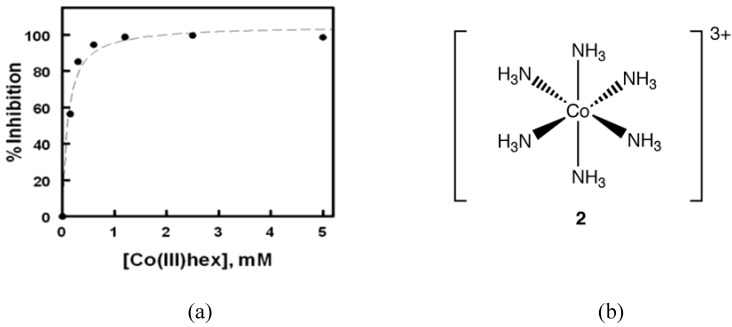
(a) Degree of inhibition of Sindbis virus plaque formation plotted as a function of Cohex concentration for the 48 h data set. The IC_50_ for inhibition was determined to be 0.10 ± 0.04 mM Cohex using a one-site dose response logistic curve fit function. (b) Structure of Cohex cation **2** (chloride counterions omitted for clarity).

In 2008, Delehanty *et al.* reported that Cohex significantly inhibited Sindbis virus replication in baby hamster kidney (BHK) cells in a dose and time-dependent manner (Figure 3) [18]. In plaque assays, the incubation of Cohex with Sindbis virus resulted in a dose-dependent decrease in virus replication when measured at both 24 and 48 hours post infection. Over the concentration range of 0 to 5 mM Cohex, the IC_50_ for the inhibition of viral replication was determined to be 0.10 ± 0.4 mM at 48 hours. Additionally, when BHK cell monolayers were pretreated with Cohex for 6 hours prior to Sindbis infection, optimal cellular morphology and plasma membrane integrity were observed at 0.6 mM to 1.2 mM Cohex. Analysis by flow cytometry confirmed that Cohex mediated a concomitant dose-dependent increase in BHK cell viability and a decrease in the percentage of Sindbis virus-infected cells (IC_50_ = 0.13 ± 0.4 mM). These findings demonstrated for the first time that Cohex possesses potent antiviral activity.

Although the detailed mechanism of Cohex activity remains elusive, certain observations as to the Cohex target in replicating viruses were made. Cohex exhibited significant antiviral activity and reduced synthesis of viral structural proteins as evidenced by EGFP reporter assays, and this information suggests that the cobalt complex inhibits viral replication via the inhibition of viral structural protein synthesis or some point further upstream in the viral replication cycle. Just as CTC-96 acts at the point of viral entry, it is possible that the high positive charge density of Cohex allows it to disrupt the interaction of Sindbis virus glycoproteins with highly negatively charged, polysulfonated heparan sulfate receptors. Another possible mechanism involves the ability of Cohex to compete with hydrated Mg(II) and interfere with virus assembly, based on the similar charge density to [Mg(OH_2_)]^2+^ [19].

**Figure 4:**
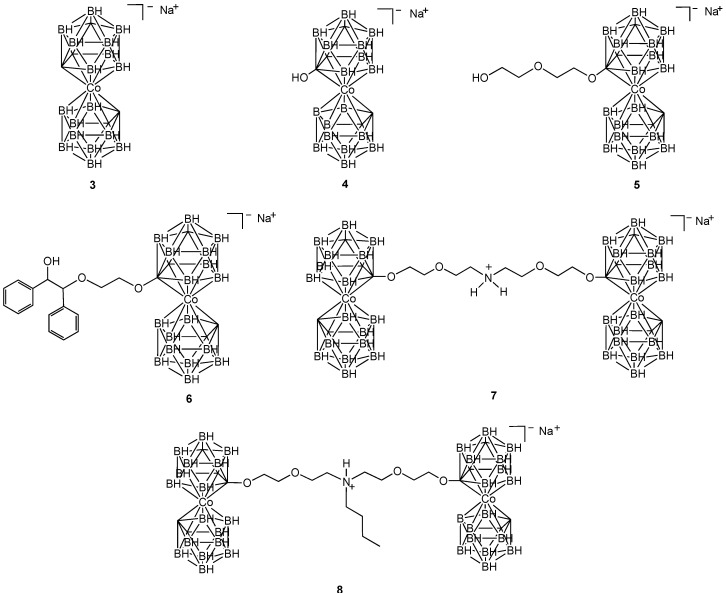
Structures of cobalt(III) carboranes used as HIV-1 inhibitors.

Carboranes are 12-vertex boron/carbon clusters, also known as dicarbollides. In 1965, Hawthorne reported a new class of cluster known as the metal bis(1,2-carbollides), formed when two dicarbollides sandwich a metal ion such as cobalt(III) [20]. Cigler and Rezacova have described the use of cobalt bis(1,2-carbollides) as antiviral therapeutics, specifically against HIV protease [21,22]. Inhibition of proteases may be achieved either chemically, or via mutation, and this is an important step in the interruption of viral maturation. Viral maturation is dependant on the protease mediated viral polypeptide cleavage. In a random testing of compound libraries, Cigler identified the cobalt bis(1,2-carbollides) as HIV protease inhibitors. Antiviral activity was analyzed using PM-1 cells infected with an HIV-1 strain. Cobalt bis(1,2-carbollides) **3**–**8** (Figure 4) were synthesized and screened in tissue cultures.

**Figure 5:**
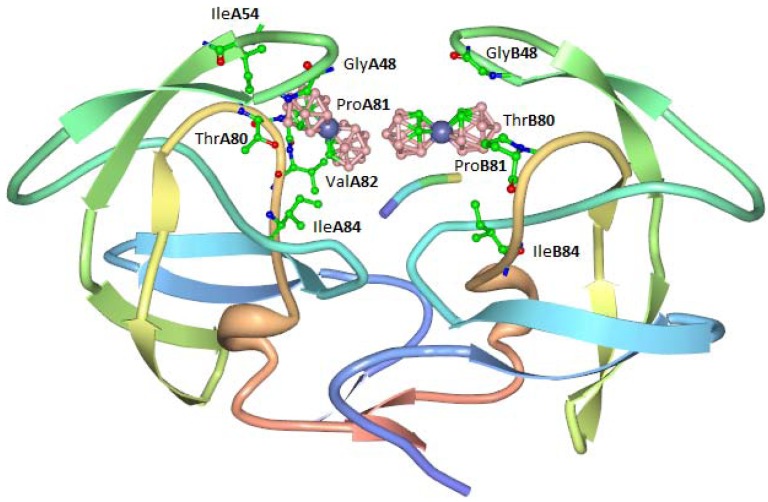
Interaction of compound **3** with hydrophobic binding pocket of HIV protease.

The highest activity was displayed by compound **8,** which exhibited an EC_50_ for inhibition of HIV-1 in tissue cultures of 250 nM (this is approximately 10× better than that observed for the related cobalt bis(1,2-carbollide) **7**. The antiviral activity of cobalt bis(1,2-carbollides) was ascribed to the formation of a stable complex with HIV protease in which the two molecules of cobalt complex bind to a hydrophobic pocket in the flap-proximal region of the S3 and S3' subsites of the enzyme. An X-ray crystal structure of the complex was obtained (Figure 5). Carboranes conjugated with porphyrins were also shown to inhibit HIV protease.

## 4. Antibacterial Activity of Cobalt(III) Complexes

A large number of reports on the antibacterial properties of cobalt complexes have appeared in the literature, with Co(II) complexes being the most studied, presumably due to their aqueous stability, availability, and ease of synthesis. However a number of examples of stable Co(III) complexes have also been reported. Although polydentate ligands with N, O and S donor atoms in the coordination sphere of cobalt are the most common ligands used to stabilize Co^3+^ ion in aqueous solution, a striking exception to this rule is the homoleptic hexammine cobalt(III) complex **2** described above which possesses high kinetic inertness in water. Reports on the antibacterial properties of cobalt(III) complexes frequently emphasize the increased effectiveness of cobalt ion coordination to a particular ligand when compared to the free ligand itself.

Complex **9,** containing a new hybrid amine-imine-oxime ligand derived from the condensation reaction of diacetylmonoxime with benzidine, was shown to be effective against *Bacillius subtilis* [23]. However the same complex showed no activity towards *Staphylococcus aureus* or the Gram-negative bacteria *Escherichia coli* and *Enterobacter fecalis*. Antibacterial activity was measured using simple zone inhibition techniques and activity was found to be less than the control antibiotics tetracycline and kanamycin (using μg of antibiotic per unit volume). Complex **9** is neutral due to the presence of one deprotonated oxime N-OH group and the oxidation state was assigned using magnetic moment measurements and absorption spectra. The related *vic*-oxime complexes **10** were described by the same authors [24]. Complexes **10a–f** all showed antibacterial activity towards *Enterobacter aeruginosa*, *E. coli, S. aureus*, and *B. subtilis niger* but were all less effective than the commonly used macrolide antibiotic azithromycin.

**Figure 6:**
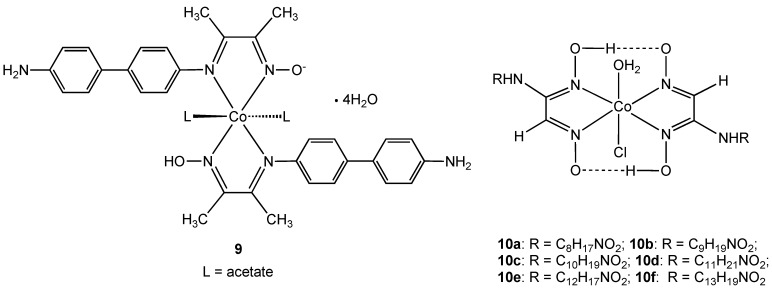
Oxime cobalt(III) complexes **9** and **10**.

**Figure 7:**
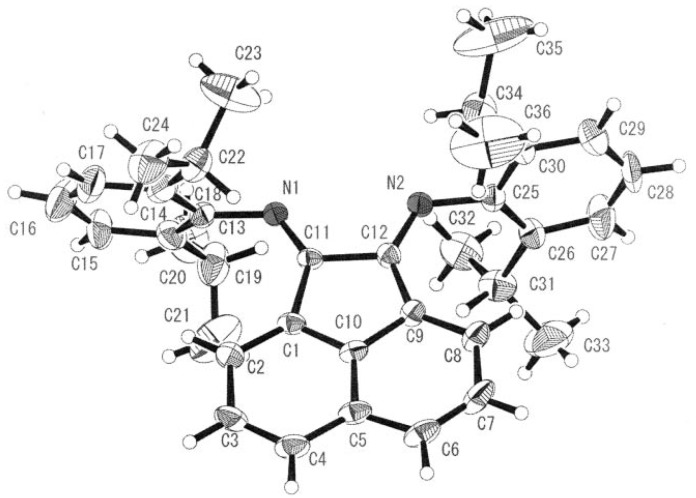
ORTEP representation of Pr-BIAN ligand (adapted from Ref. [25]).

A cobalt(III) complex containing the rigid bidentate nitrogen ligand *bis*[*N*-(2,6-diisopropyl-phenyl)imino]acenaphthene (Pr-BIAN) demonstrated potent antibacterial activity towards *S. aureus* and *E. coli* (Figure 7) [25]. The authors noted the observed antimicrobial activity was highly dependant on the bulkiness of the *N*-bisimine derivatives which increases the relative lipophilicity of the molecule. Chelation of a bulky ligand to a metal cation reduces the polarity of the ion due to ligand orbital overlap with the metal orbitals resulting in a delocalization of positive charge. An increase in lipophilicity of a metal complex enhances bacterial cell membrane penetration and blocking of metal binding sites on enzymes. Coordination of the *N*-bisimine ligand to Co(II) instead of Co(III) had no appreciable effect on the potency of the complex supporting the hypothesis that hydrophobic shielding of the metal center by the chelating ligand is responsible for bioactivity.

**Figure 8:**
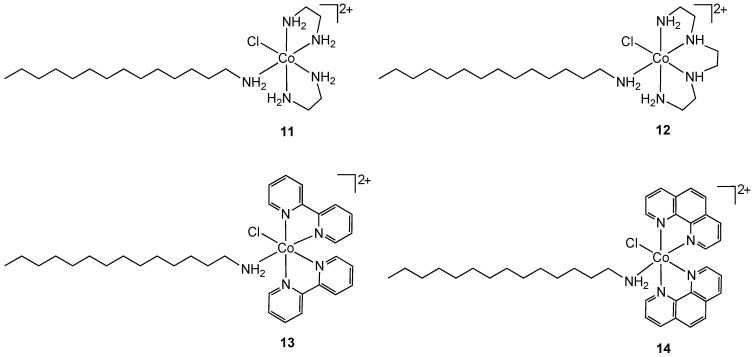
Cobalt(III) containing metallosurfactants.

Another approach for improving the lipophilicity of metal complexes can be achieved by functionalization with surfactant-like ligands. Arunachalam reported the synthesis of metallosurfactant complexes of Co(III) containing ethylenediamine (**11**), triethylenetetramine (**12**), 2,2'-bipyridyl (**13**) and 1,10-phenanthroline (**14**) nitrogen chelates (Figure 8) [26]. The surfactant properties of the complexes were provided by a coordinated C-14 long chain amine.

**Figure 9:**
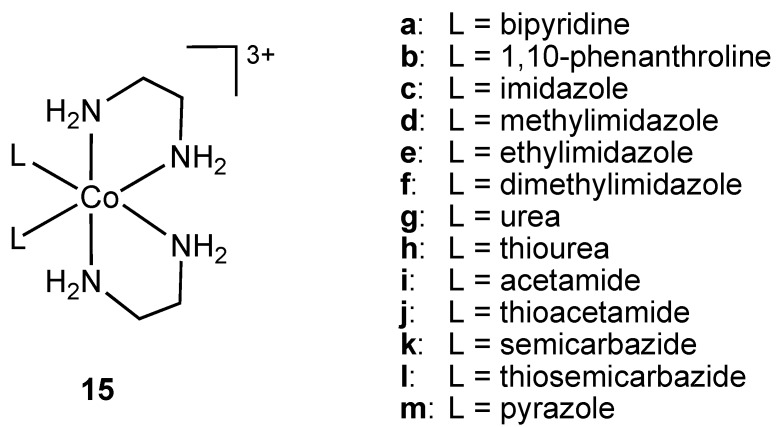
Ethylenediamine cobalt(III) cations **15** (bipyridine and 1,10-phenanthroline ligands are coordinated in bidentate chelating mode to the metal ion).

Complexes **11**–**14** were screened for antibacterial properties and activities compared with the antibiotic ciprofloxacin. All showed considerable activity against the Gram-positive bacteria *S. aureus* and *B. subtilis* and the Gram-negative bacteria *E. coli* and *P. aeruginosa* but did not achieve the same effect as ciprofloxacin. The authors did not compare the activities of **11**–**14** to non-surfactant containing cobalt(III) analogs so it is difficult to reach conclusions about the mode of action. The hydrophobicity of the complexes can result in increased damage to bacterial cell walls. Complex binding to DNA was also suggested as a possible mode of action [26]. Nagababu and co-workers screened a large number of bis(ethylenediamine)cobalt(III) cations **15** (Figure 9) against Gram-positive and Gram-negative bacteria (*E. coli*, *E. coli* HB101, *Salmonella typhimurium*, *Proteus vulgaris*, *P*. *aeruginosa*, *S*. *aureus*, *S*. *faecalis*, *B*. *subtilis*) [27]. 

The complexes were synthesized via chloride displacement from *trans*-[CoCl_2_(en)_2_] (en = ethylenediamine) and subsequently characterized using visible absorption, IR and ^1^H-NMR spectroscopies. Antibacterial activities of **15** were determined using zone inhibition, minimum bactericidal concentration (MBC) and time period of lethal action. Cations **15a** and **15b** containing bidentate, chelating ligands were shown to possess the highest potency against *E. coli*. The 1,10-phenanthroline containing cation **15b** had higher activity than the antibiotics ampicillin, streptomycin, gentamicin, chloramphenicol and ciprofloxacin [27].

**Figure 10:**
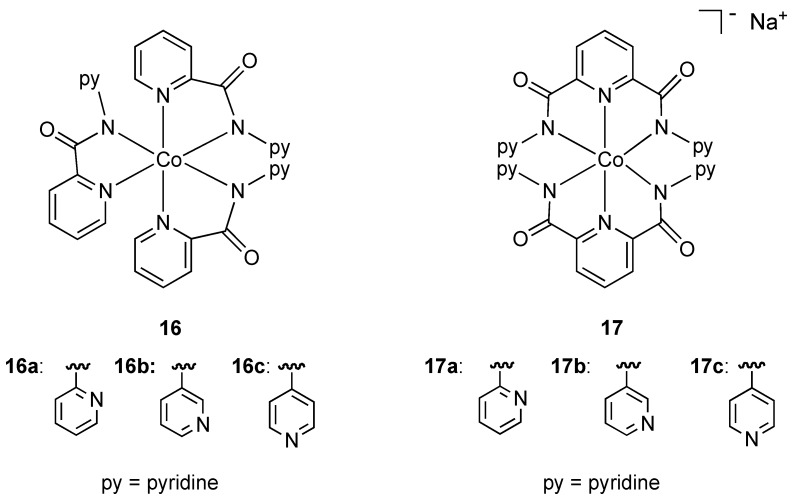
Neutral and anionic pyridine-amide cobalt(III) complexes.

In an effort to increase the hydrophobic ligand shell around octahedral cobalt(III) and consequently improve membrane diffusion properties, Mishra and coworkers prepared the neutral and anionic mixed pyridine-amide complexes **16** and **17** (Figure 10). All complexes contained the ligand binding through nitrogen donor atoms in a chelating fashion. The neutral anionic complexes **16** and **17a** showed strong activity against resistant strains of *Pseudomonas*, *E. coli*, and standard strains of *Shigella* and *Klebsiella* while the anionic complexes **17b** and **17c** were also found to have activity against *Pseudomonas*. The authors suggest that the presence and position of uncoordinated pyridine rings may be responsible for antibacterial properties. To the best of our knowledge these are the only examples of *anionic* cobalt(III) complexes (**17a–c**) being used as bactericides.

**Figure 11:**
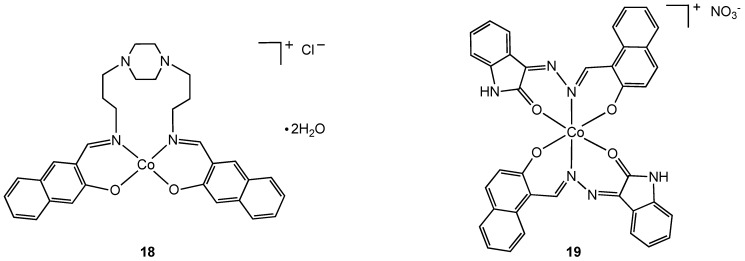
Tetragonal and octahedral cobalt (III) complexes containing N, O chelating Schiff base ligands.

A number of cobalt(III) complexes containing N,O chelates have been shown to have antibacterial properties [28,29,30,31]. An uncommon example of a tetragonal cobalt(III) complex (**18**) was isolated from the reaction of Schiff base 1,4-bis[3-(2-hydroxy-1-naphthaldimine)propyl]piperazine (bappnaf) with cobalt(II) chloride in hot methanol (Figure 11). Evidence for oxidation of cobalt(II) to cobalt(III) in this reaction was provided by elemental analysis, molar conductivity, thermal gravimetric analysis and mass spectroscopy [31]. The related complex **19** was prepared directly from a cobalt(III) precursor, [Co(NH_3_)(CO_3_)]NO_3_•0.5 H_2_O, via ligand displacement [29,30] and screened for antibacterial properties. Complexes **18** and **19 ** exhibit broad-spectrum antibacterial activity with the metal complexes having higher activities than the free ligand. Enhanced lipophilicity due to metal coordination and chelation theory is used to explain the increase in bioactivity although clearly there may be other contributing factors.

**Figure 12:**
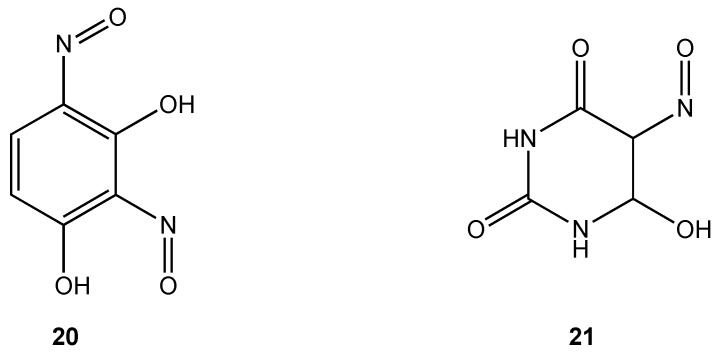
Dinitrosoresorcinol and violuric acid ligands.

Only two cobalt(III) complexes containing O, O chelates have demonstrated antibacterial properties. Cobalt(III) complexes derived from dinitrosoresorcinol (**20**) and violuric acid (**21**) (Figure 12) were synthesized by direct reaction of the ligands with cobalt(II) chloride in ethanol in varying molar ratios [32]. Biocidal activity against *Serratia* sp., *Bacillus stearothermophilus*, *B. subtilis* and *Pseudomonas* sp. was demonstrated to be higher for the metal complexes compared to either the free ligand or simple metal salts.

**Figure 13:**
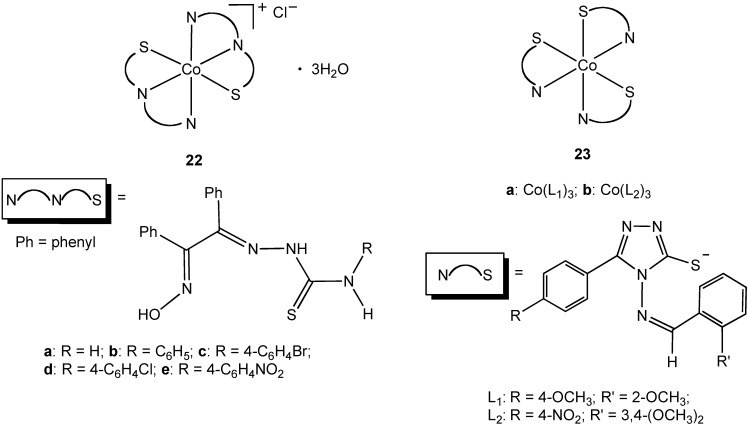
Cobalt(III) complexes containing bidentate and tridentate nitrogen/sulfur donor ligands.

Complexes containing a sulfur atom as part of a ligand donor set represent the largest class of cobalt(III) compounds studied for their antibacterial properties [33,34,35,36,37,38,39]. Sharma and coworkers synthesized octahedral Co(III) complexes **22a–e** containing tridentate N, N, S thiosemicarbazone ligands (Figure 13) [35]. The thiosemicarbazones were of prior interest due to their extensive pharmacology including carcinostatic potency, antitumor, antibacterial, antiviral, antifungal, antimalarial, and antiamoebic properties. Complexes **22a–e** proved to be toxic to *E. coli* and *S. aureus* with an increase in potency compared to the free thiosemicarbazone ligands [35]. All of the complexes studied had less activity when compared to streptomycin. Complexes **23a–b** showed moderate broad-spectrum antibacterial activity [34]. The synthesis of **22** and **23** all required the use of hydrogen peroxide as an oxidant to convert Co(II) to Co(III), an alternative approach to the use of a pre-formed Co(III) precursor.

**Figure 14:**
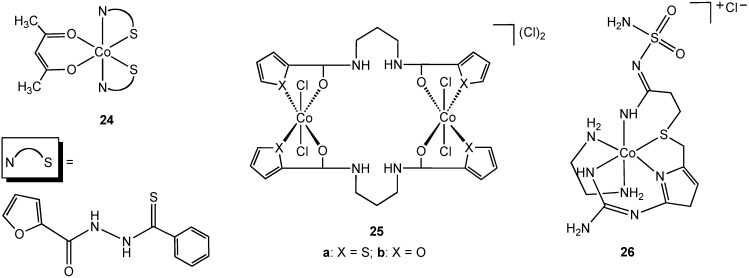
Cobalt(III) complexes containing bidentate and tetradentate nitrogen/sulfur donor ligands.

Singh reported the synthesis and characterization of the N,S chelate cobalt complex **24** derived from a 2-furanthiocarbohydrazide ligand (Figure 14) [39]. The complex is octahedral, two N, S ligands occupying four coordination sites in the complex, the fifth and sixth sites are completed with an acetylacetonate ligand. Due to the lack of solubility of **24** in water antibacterial screening using zone inhibition was performed using DMSO solution (blank DMSO/H_2_O was used as a control). Complex **24** is active against *Staphylococcus albus* and *Enterobactor*. The free 2-furanthiocarbohydrazide ligand possesses no antibacterial activity. The bimetallic cobalt (III) complex **25a** (Figure 14) was shown to have antibacterial activity against *E. coli*, *Yersinia enterocolitica*, *Bacillus magaterium*, and *B. subtilis*. Surprisingly, the furan form of the complex (**25b**), containing an oxygen donor atom in place of sulfur, demonstrated no activity towards the same group although no explanation was offered for this difference in activity.

Famotidine is a sulfur containing tetradentate N, N, N, S ligand and is a histamine H_2_-receptor antagonist. In addition to being widely used in the treatment of peptic ulcers and reflux esophagitis, famotidine has also been used in the treatment of Zollinger-Ellison syndrome, Parkinson's disease and Alzheimer's disease. In 2006, Miodragović described the coordination of famotidine to cobalt(III) and reported the isolation of complex **26** (Figure 14) [36]. The octahedral geometry and tetradentate ligand coordination was confirmed using X-ray crystallography in which extensive intermolecular hydrogen bonding and non-covalent CH•••π and NH•••π interactions, as well as π-stacking interactions were observed. Complex **26** has antibacterial activity against *E. coli*, *S. aureus* and *Micrococcus lysodeikiticus*, showing better growth inhibitory activity in comparison with famotidine alone.

Only two selenium containing cobalt(III) compounds have shown antibacterial activity [40]. Complexes **27** and **28** in which the pyridine and quinoline derived ligands coordinate to cobalt via nitrogen and selenium donor atoms were synthesized and characterized using X-ray crystallography. Both complexes exhibited activity towards *E. coli*, *B. subtilis, S. aureus* and *Micrococcus lysodeikiticus*.

**Figure 15:**
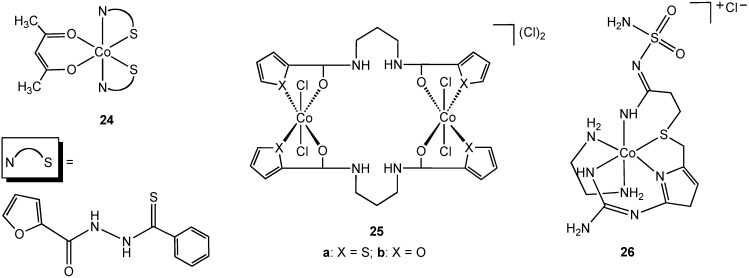
Selenium containing and organometallic cobalt(III) complexes.

Organometallic complexes containing a formal metal carbon bond are rare in biology, with vitamin B_12_ representing a well-known example of this class of molecule. To the best of our knowledge, only one class of cobalt(III) containing organometallic complex has been studied with respect to chemotherapy. Navaneetha prepared the series of organometallic methyl cobalt(III) complexes **29a**-**e** in which the ligand *trans* to the -CH_3_ ligand was varied [41]. All five complexes were active against *Klebsiella pneumoniea*, *S. aureus*, *E. coli* and *B. subtilis*.

## 5. Toxicity of Cobalt(III) Complexes

Cobalt is generally not considered to be a very toxic element. Most toxicity studies have been concerned with Co(II) metal ions, surgical implants, or cobalt metal dust [42], with one notable example of cobalt-induced mortality from drinking large quantities of beer that contained cobalt chloride or cobalt sulfate as a foam stabilizer (“beer drinker's cardiomyopathy”) [43]. There is much less known about toxicity of Co(III) complexes, with Cohex being the most studied. In general, it does not appear that Co(III) complexes are toxic at moderate levels of exposure, although some adverse effects on kidney function were reported.

The toxicity of metal-ion based therapeutics is a concern due to the intrinsic toxicity of some metal ions themselves. However, there are no open coordination sites on Cohex available for interacting with the environment. Further, the complex sequesters the inner-sphere ammonia ligands from most exchange-reactions in solution; therefore, interactions with solution molecules are by "outer-sphere" coordination via water bridges to the ammonia ligands and via the high charge-density of the Co(III) ion [44,45]. Due to the kinetic inertness of Co(III), we believe that the compound is stable such that its biological interaction will also reflect these modes of interactions. Thus, the toxicity of Cohex cannot be inferred from that of the Co^3+^ ion itself.

**Figure 16:**
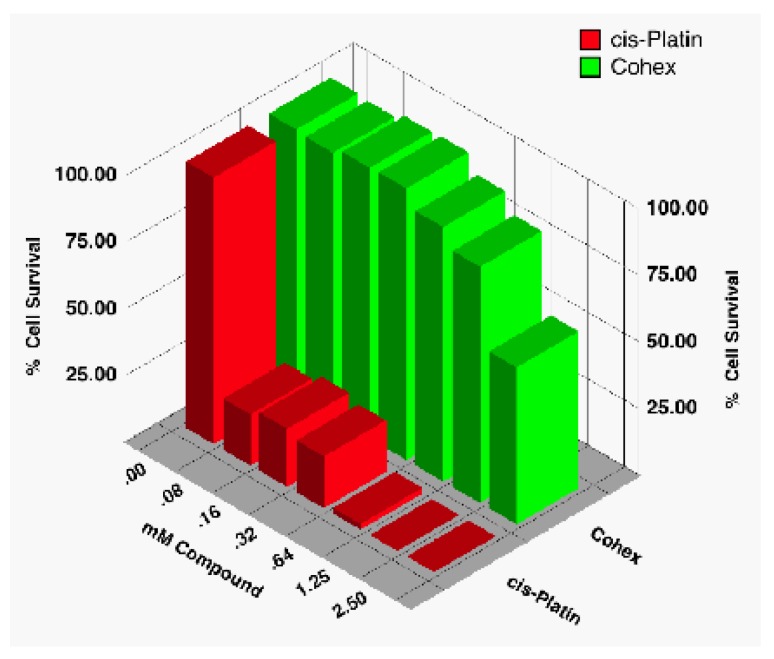
Comparison of cytotoxicity of Cohex against BHK cells with commercial drug cisplatin using CellTiter96.

There are only a few sources dealing with the toxicity of Cohex. In our laboratory, we have found the compound to have low to moderate toxicity [18], much less so than, say, cisplatin. In an early study, we assayed for the cytotoxicity of Cohex against BHK cells using CellTiter96® Proliferation Assay (Promega, Madison, WI) and compared it to that of cisplatin (Figure 16). 

As can be seen, Cohex cytotoxic effects are only evident around 1 mM or higher, whereas cisplatin was toxic even at the lowest concentration tested (80 μM). Since that study, we have routinely used up to 2.5 to 5 mM of compound in our flow cytometry studies without detecting a significant amount of cell death (Table 1). In these studies, we measured the state of the cell (up to 10^4^ cells) using propidium iodide for cell viability. Studies in other laboratories generally seem to confirm that toxicity starts around 1 mM but never seems to fully kill the cells—even at concentrations as high as 5 mM (unpublished data from this laboratory and private communications).

**Table 1:** Typical flow cytometry results for uninfected BHK cells.

mM Cohex	0.00	0.60	1.25	2.50	5.00
% Live	75.4	73.6	74.8	78.2	73.5

Beyond the cytotoxicity studies, only two published reports exist of the toxicity of Cohex in small animal models (mice). An earlier paper reported on the effects of Cohex on oxidative stress-related parameters in male albino BALB/c mice [46]. Mice were divided into four groups consisting of 6–8 animals each and one group served as the control. The other three groups of mice were administered Cohex in drinking water at concentrations of 25, 50, and 100 ppm for a period of 14 weeks. They found that tissue levels of the compound followed the trend kidney > liver > intestine > blood > spleen > lungs. Cobalt content (μg/g tissue) increased from 2.172 to 5.970 in kidney, 4.443 to 7.152 in liver, 1.571 to 2.828 in intestine, 0.305 to 0.504 in spleen, 1.289 to 1.455 in lung, and 0.286 to 1.185 (μg/mL) in blood with respect to a control. The concentrations of Cohex used for their experiments were apparently nontoxic since no treatment-related changes in daily food intake or growth rate were observed.

In a follow-up study on the toxicity of the compound at higher dosages, Naura and Sharma [46] administered intraperitoneal injections once daily for 3 consecutive days at 5, 10, 20 mg/kg body weight and sacrificed the mice (male albino BALB/c) 24 h after last injection. They observed accumulation of Co in kidney > liver > spleen > lungs, with the increase of cobalt from 2.712 to 14.332 in kidney and 4.443 to 10.637 μg/g in liver tissues. In blood, the increase was from 1.269 to 1.729 μg/mL in blood when the dose was raised from zero to 20 mg/kg of body weight. Urea and creatine were observed to increase in the blood of the treated mice suggesting that Cohex can affect the normal functioning of the kidney following acute administration. However, none of the mice died before being sacrificed.

**Figure 17:**
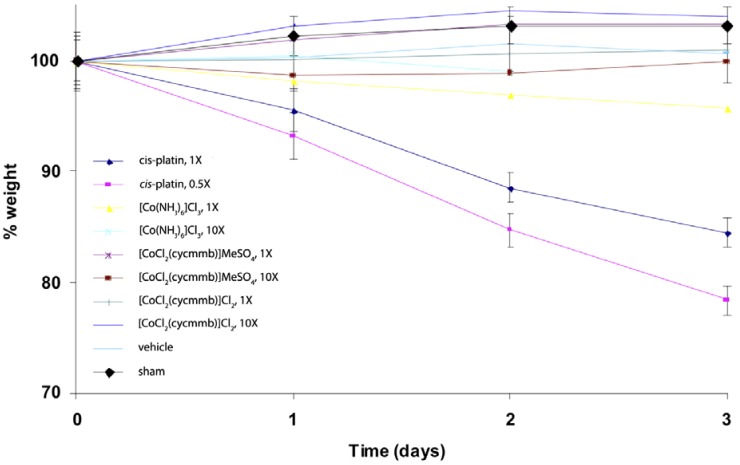
Weight loss of C57BL/6J mice exposed to platinum(II) and cobalt(III) complexes. 1X is equivalent to approx. 1 mM [Co(NH_3_)_6_]Cl_3_ in blood volume of the mice. Cycmmb = methyl 4-[(1,4,7,10-tetraazacyclododec-1-yl)methylbenzoate.

We have also done some studies of Cohex toxicity in mice. A preliminary mice toxicity study showed that similar results as the cytotoxicity work (Figure 17) [47]. For this study, mice were administered the agents at doses of 0.5, 1, 3 and/or 10X, where 1X is the molar equivalent of the LD_50_ of cisplatin. Administration was by inter-peritoneal injection under light isoflurane anesthesia. The different compounds were dissolved in water at the desired concentration and all animals received 1 mL injection volume as a bolus. Vehicle shams received a 2 mL injection of water. Mouse weights were monitored daily, as were behavioral signs. A rough calculation indicates at 1× concentration for Cohex is about 1 mM Cohex in the blood volume.

Toxicity for the CTC series of cobalt complexes were briefly mentioned [9] in a 1998 report stating that all the CTC complexes tested were non-toxic to Vero cells upon continuous exposure to ≤ 25 μg/mL, whereas 50 μg/mL of CTC 96, nine times daily, did not irritate uninfected rabbit eyes, but cytotoxicity was evident at ≥ 50 μg/mL. Cigler, *et al.* [21] reported on the potent, specific, and selective inhibition of HIV protease by parental and substituted metallacarboranes, specifically, cobalt bis(1,2-dicarbollides). Antiviral activity was analyzed by using PM-1 cells infected with HIV-1 strain NL4-3 modified from a published procedure ref. PM-1 cells were infected by co-culture and washed 4 h after infection, and compounds **3**–**8** or the solvent DMSO, respectively, was added after the wash. No significant toxicity of tested compounds in tissue cultures was observed in the concentration range up to 50 μM. Mishra, *et al.* [48] studied several pyridine-amide based cobalt(III) complexes for their antimicrobial activity. Cytotoxic activity of the metal complexes was also determined via growth inhibition by MTT assay. The final concentrations ranged from 1,500–0.73 μg/mL. They found that cobalt(III) complexes were less cytotoxic than that of gentamycin. The cytotoxic activity results suggested that these cobalt complexes were less cytotoxic at these moderate concentrations.
